# Supplementary figures and images for: Sexual differences in neuronal and synaptic properties across subregions of the mouse insular cortex
Source: Biol Sex Differ. 2024 Apr 1;15:29. doi: 10.1186/s13293-024-00593-4 (PMC10983634; doi:10.1186/s13293-024-00593-4)

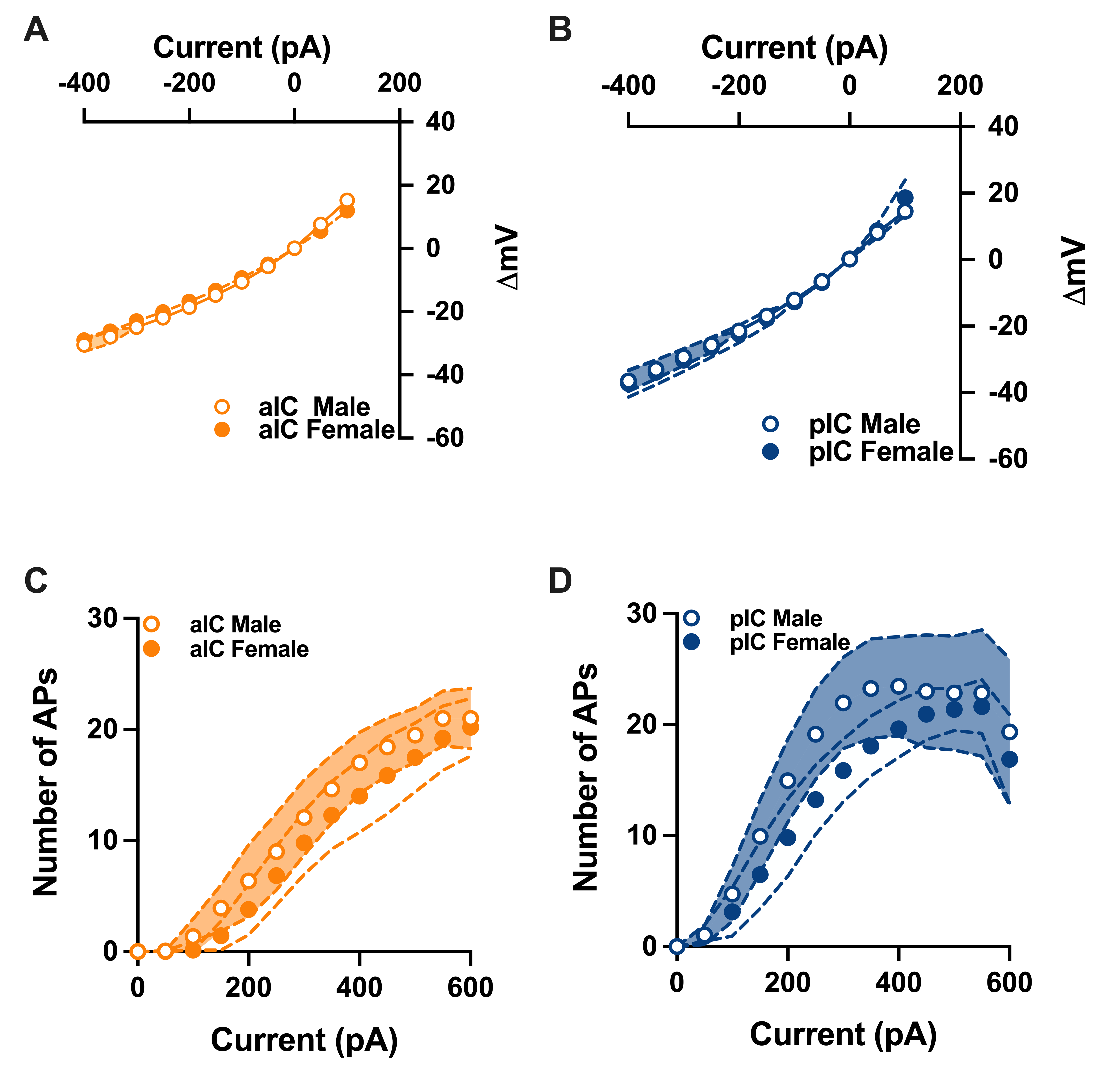

Supplement: Supplementary file 1 — Additional file 1: Figure S1. Comparative analysis of I-V relationships and excitability in male and female anterior and posterior IC neurons. (A-B) Incremental current injections of 50 pA from − 400 pA to + 50 pA exhibited no significant differences in the I–V relationship for male and female neurons in both anterior and posterior IC. (C-D) Gradual depolarizing current injections (500 ms, ranging from 0 to 600 pA in 50 pA increments) revealed comparable excitability in both male and female neurons within the same area. (A-B) Each dot similarly represents the group mean value at the given current step, with data shown as mean ± SEM in an XY plot. A Mann–Whitney U test was used, and *p-value < 0.05 was considered significant. (C-D) Each dot indicates the group mean value for the respective current step, with data presented as mean ± CI in an XY plot. A Mann–Whitney U test was used for statistical analysis, with a *p-value < 0.05 considered significant. (A-D) aIC male is represented as 10/14 in dark orange, aIC female as 6/14 in light orange, pIC male as 15/20 in dark blue, and pIC female as 12/16 in light blue. [file 13293_2024_593_MOESM1_ESM.tiff]

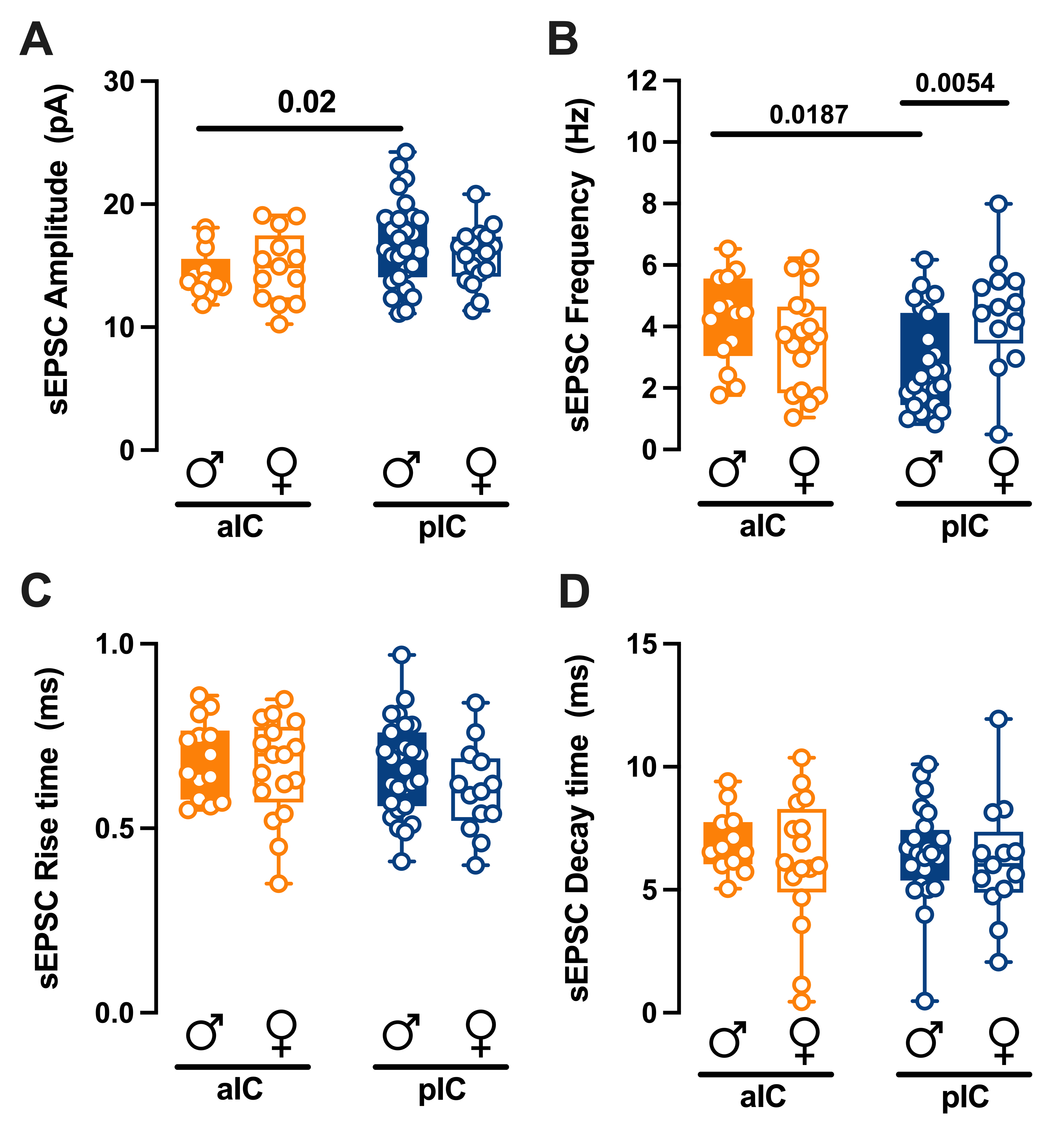

Supplement: Supplementary file 2 — Additional file 2: Figure S2. Quantitative evaluation of amplitude, frequency, and kinetics of excitatory events in adult male and female anterior and posterior IC neurons. A quantitative analysis of mean amplitude and frequency in relation to area and sex demonstrated greater amplitude (A) but lower frequency (B) of pIC excitatory events compared to aIC events in adult males only. (B) In females, pIC was characterized by higher excitatory transmission compared to male pIC. (C-D) The kinetics of sEPSC were consistent across both areas and sexes. (A-D) Individual neurons are represented by single dots. Data are displayed as box and whisker plots (min., max., median). A two-way ANOVA followed by a Šídák multiple comparison test was used for data analysis. P-values < 0.05 are indicated in the graphs. aIC male is represented as 9/14 in dark orange, pIC male as 16/27 in dark blue, aIC female as 6/13 in light orange, and pIC female as 12/17 in light blue. [file 13293_2024_593_MOESM2_ESM.tiff]

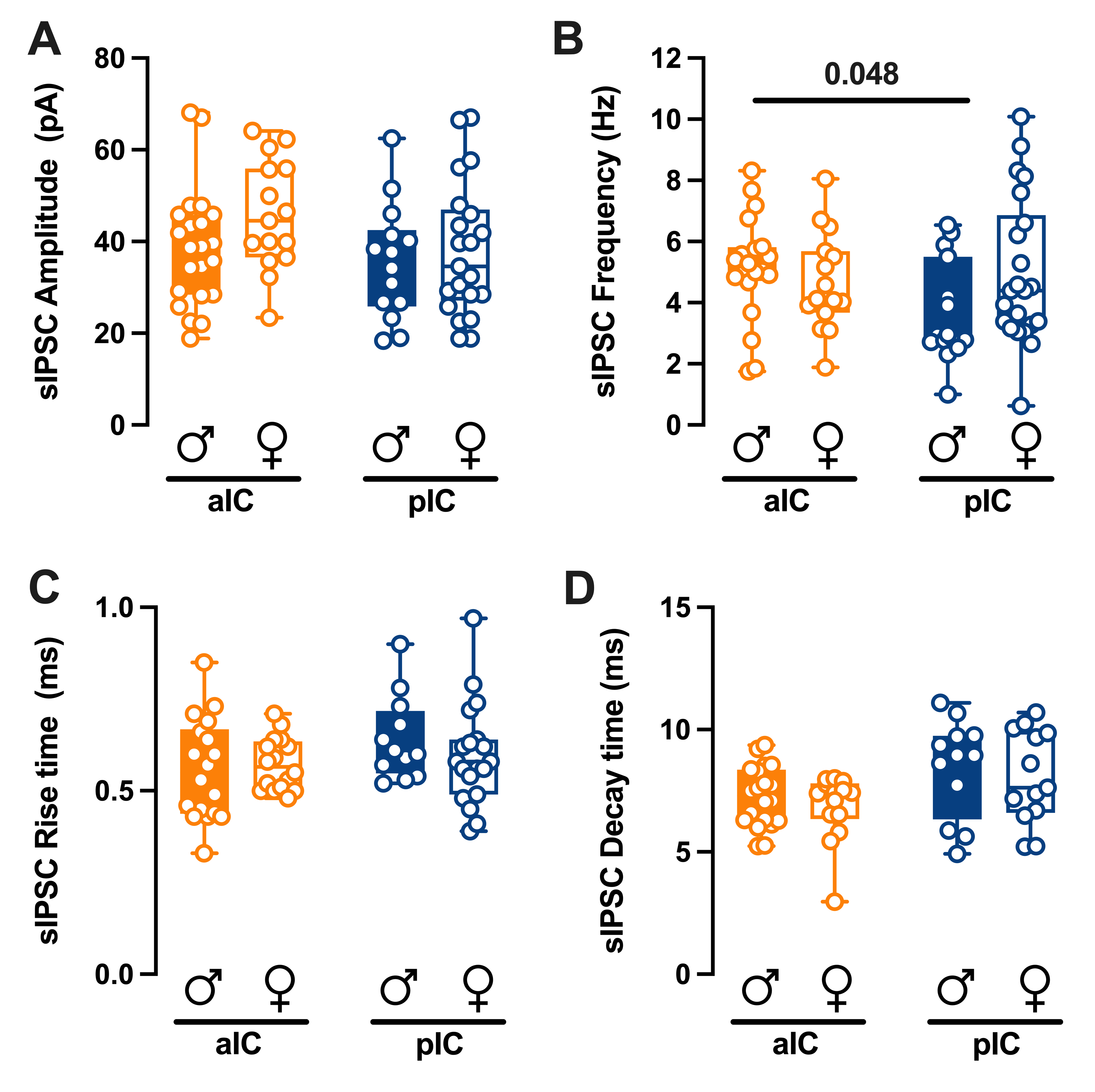

Supplement: Supplementary file 3 — Additional file 3: Figure S3. Quantitative assessment of sIPSC events in male and female anterior and posterior IC neurons: inhibitory transmission comparison. A quantitative analysis of sIPSC events in relation to area and sex showed similar amplitude of sICPCs in both males and females (A), while there was a lower frequency of inhibitory events in the pIC when compared to the aIC, in only male (B). No differences were found in the kinetic of sIPSCs (C-D). Each dot represents an individual neuron. Data are displayed as box and whisker plots (min., max., median). A two-way ANOVA followed by a Šídák multiple comparison test was used for data analysis. P-values < 0.05 are indicated in the graphs. aIC male is represented as 10/21 in dark orange, pIC male as 11/15 in dark blue, aIC female as 10/16 in light orange, and pIC female as 11/19 in light blue. [file 13293_2024_593_MOESM3_ESM.tiff]
